# Examining the Effectiveness of a Clinical Skills Curriculum: A Critical Analysis

**DOI:** 10.7759/cureus.99479

**Published:** 2025-12-17

**Authors:** Christian Raphael, Thuraya HajAli, Sarine Sarkis

**Affiliations:** 1 Anesthesiology and Pain Medicine, American University of Beirut Medical Center, Beirut, LBN

**Keywords:** active learning, clinical skills, competency-based curriculum, medical education, teaching in medicine

## Abstract

The clinical skills curriculum for first- and second-year medical students at the American University of Beirut is a key component of the "Impact Curriculum," a competency-based framework introduced in 2013. It promotes active learning, teamwork, critical thinking, and self-reflection through small-group sessions focused on history taking, physical examination, communication, and clinical reasoning. Assessments include Objective Structured Clinical Examinations (OSCEs), Multiple-Choice Questions (MCQs), and real-time feedback. Strengths include clear objectives, diverse teaching methods, and early interprofessional exposure. Challenges involve improving training in abnormal findings, aligning with organ-based content, managing OSCE stress, and enhancing faculty support. Continuous refinement ensures graduates deliver high-quality, patient-centered care.

## Introduction

Imagine learning to ride a bike. There's an end goal, being able to ride the bike independently, but the focus is on the process of learning how to balance, pedal, and steer. A clinical skills course is similar, with the desired outcome being competent clinical practice, but the focus is on the process of developing the necessary skills through practice and feedback [1]. A clinical skills curriculum for medical students is a critical component of their education, shaping their abilities to perform in real-world medical settings. A comprehensive analysis of such a curriculum must consider various pedagogical approaches and their impact on the development of clinical competencies. The following is an analysis of the clinical skills course taught to first- and second-year medical students at the American University of Beirut in Lebanon, as part of their four-year medical curriculum called the Impact Curriculum. The aim of this analysis is to critically evaluate the course's structure, pedagogical foundations, assessment strategies, strengths, and limitations and to determine how effectively the curriculum prepares students for clinical practice while identifying areas for future improvement.

## Technical report

The Impact Curriculum

**Figure 1:**
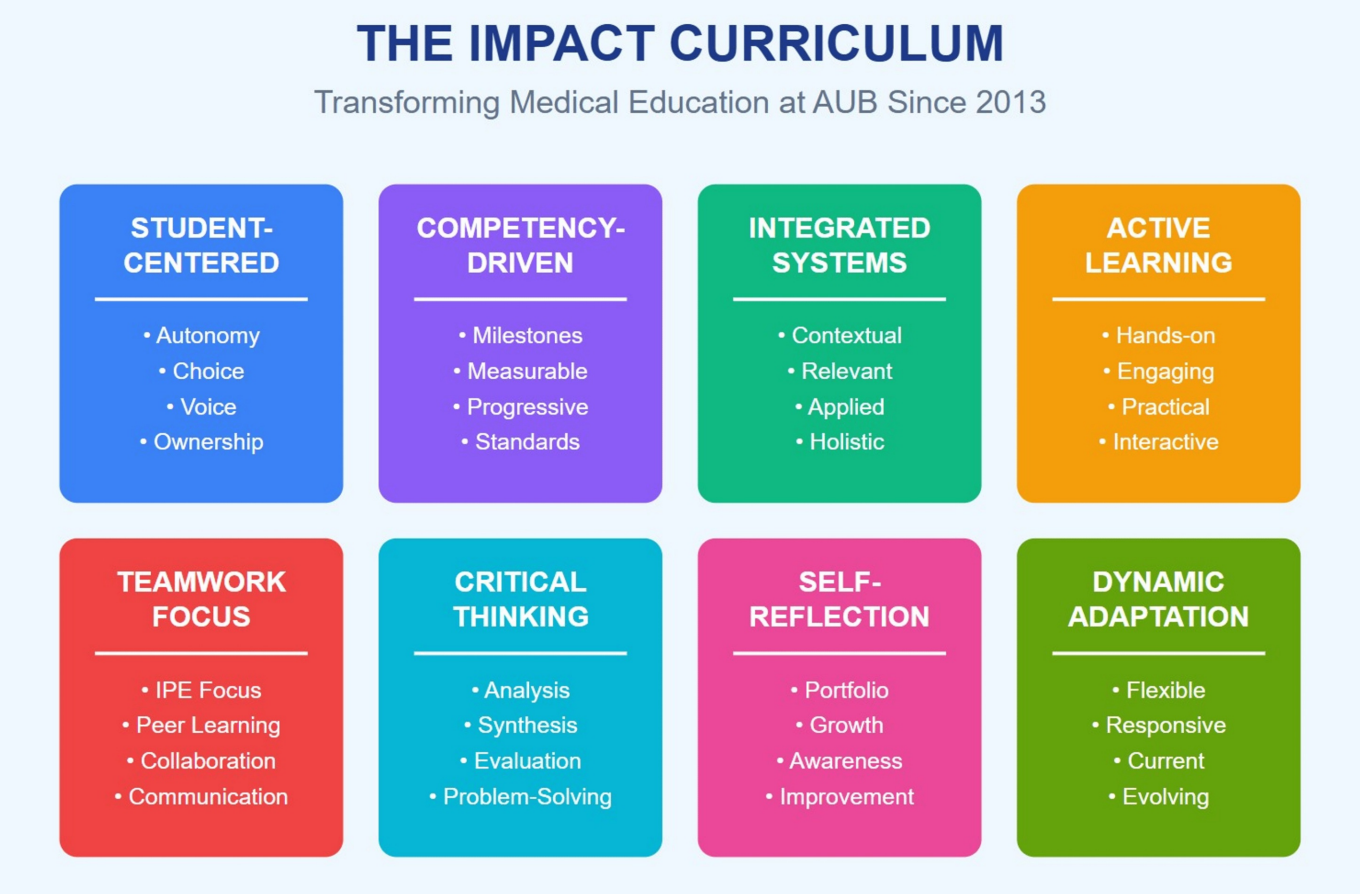
The Impact Curriculum framework (2013-present) Creating competent, compassionate, and adaptive physicians. AUB: American University of Beirut; IPE: interprofessional education This image was created by the authors of this study.

**Figure 2:**
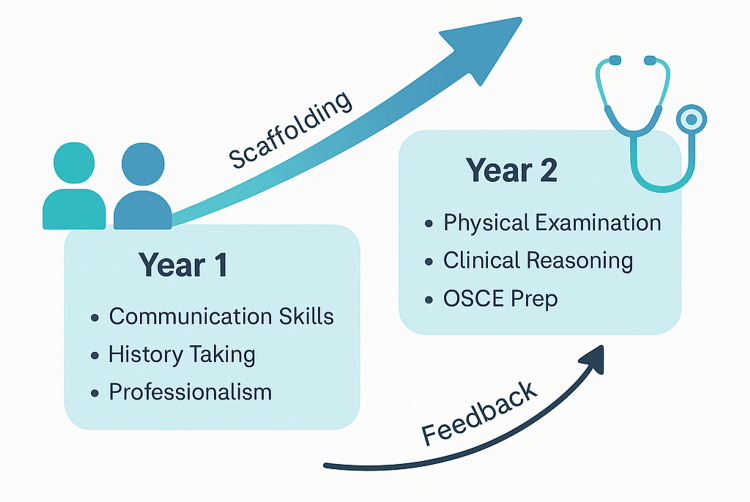
Structure of the clinical skills course within the Impact Curriculum OSCE: Objective Structured Clinical Examination; Prep: preparation This image was created by the authors of this study.

**Figure 3:**
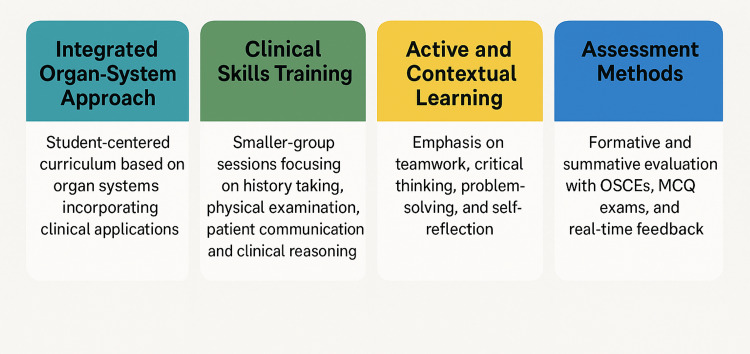
Impact Curriculum overview OSCEs: Objective Structured Clinical Examinations; MCQ: Multiple-Choice Question This image was created by the authors of this study.

In 2013, this new curriculum came to replace the traditional medical school curriculum (Figure 1). The Impact Curriculum is designed as a student-centered, competency-driven, integrated, and dynamic educational framework that prioritizes active and contextual learning, teamwork, critical thinking, problem-solving, and self-reflection. Over the initial two pre-clinical years, it adopts an integrated organ system-based approach heavily infused with clinical applications, including exposure to real or simulated patient encounters (Figure 2 and Figure 3). This early contextual exposure strengthens students' ability to transfer theoretical knowledge to authentic clinical situations, thereby supporting the development of clinical reasoning and diagnostic accuracy in later years.

A key component of this approach involves small-group clinical skills sessions, providing hands-on training in history taking, physical examination, patient communication, and clinical reasoning. The small-group format is intentionally designed to promote individualized feedback, refine psychomotor and communication skills, and foster professional behaviors in a controlled learning environment. These sessions progress in complexity and emphasize repetitive, deliberate practice to ensure skill mastery, a structure that directly supports the attainment of competency-based outcomes such as proficiency in core examination techniques and confidence in patient interactions.

Assessment encompasses both formative and summative approaches, incorporating real-time feedback from instructors and peers, as well as Objective Structured Clinical Examinations (OSCEs) and Multiple-Choice Question (MCQ) exams. This multimodal assessment strategy is aligned with the course's learning outcomes by ensuring the continuous monitoring of skills acquisition, evaluating decision-making and clinical reasoning, and verifying students' readiness to perform safely in clinical settings.

By the end of the course, students are expected to demonstrate good practical abilities and professional behavior, communicate effectively with patients using both verbal and nonverbal cues, and understand the main components of a medical interaction. They should be able to explore a patient's main complaint, take a detailed medical history, and accurately complete a patient-oriented medical record. Students will also learn to perform basic general and organ-specific physical examinations on adult patients, use foundational clinical reasoning skills, and apply these skills to analyze and diagnose different medical conditions. Each structural element of the curriculum, such as integrated content, experiential learning, small-group instruction, and multimodal assessment, has been deliberately selected to support the achievement of these specific educational outcomes.

The importance of a clinical skills course

Medical education has undergone significant changes in recent decades due to various factors, including technological advancements and pressure from stakeholders such as patients, healthcare institutions, regulatory bodies, and medical professionals. Many medical school curricula have shifted from traditional, teacher-centered approaches to student-centered learning methods, aiming to equip future doctors with the skills and competencies needed for continuous improvement and patient-centered care. This shift is reflected in competency frameworks like the Canadian Medical Education Directives for Specialists (CanMEDS), established by institutions such as the Royal College of Physicians and Surgeons of Canada, as well as counterparts in the United States and the United Kingdom. These frameworks emphasize competency-driven learning across various domains of medical practice, including communication, collaboration, leadership, advocacy, scholarship, and professionalism, with the goal of enhancing medical care [1].

Starting clinical rotations can be stressful for medical students. This is because they must deal with many new things at once, such as understanding their new roles and responsibilities, working with teams in clinical settings, managing new schedules and workloads, applying their knowledge from pre-clinical courses to real patients, and performing new clinical skills. In fact, teachers in charge of these clinical rotations often find that medical students are not fully prepared for these experiences [2]. Moreover, learning in small groups fosters a more profound comprehension of the subject matter, improves problem-solving abilities, promotes active participation, and cultivates team communication skills that are relevant throughout one's medical career. Having medical students practice clinical skills is a great way to reduce their anxiety and boost their confidence before starting clerkships. Research shows that the amount of procedural training students get in later years can vary widely [3], leading to ongoing anxiety and a lack of skill in performing procedures. Therefore, our course ensures all students have a baseline level of competency before starting their clinical rotations. So, what necessitates the development of a distinct pre-clinical curriculum? Real clinical experiences are vital, but the traditional clerkship model may not fully meet students' learning needs, prompting the need for supplementary curricula. The specific changes introduced in the new clinical skills curriculum at the University of Calgary were designed by Veale et al. to address the learning gaps in the existing clerkship model. The introduction of the new clinical skills curriculum led to significantly higher performance in clerkship evaluations and the Medical Council of Canada Qualifying Examination (MCCQE) Part 1 exam [3].

Interplay of curricula in the clinical skills course

**Figure 4:**
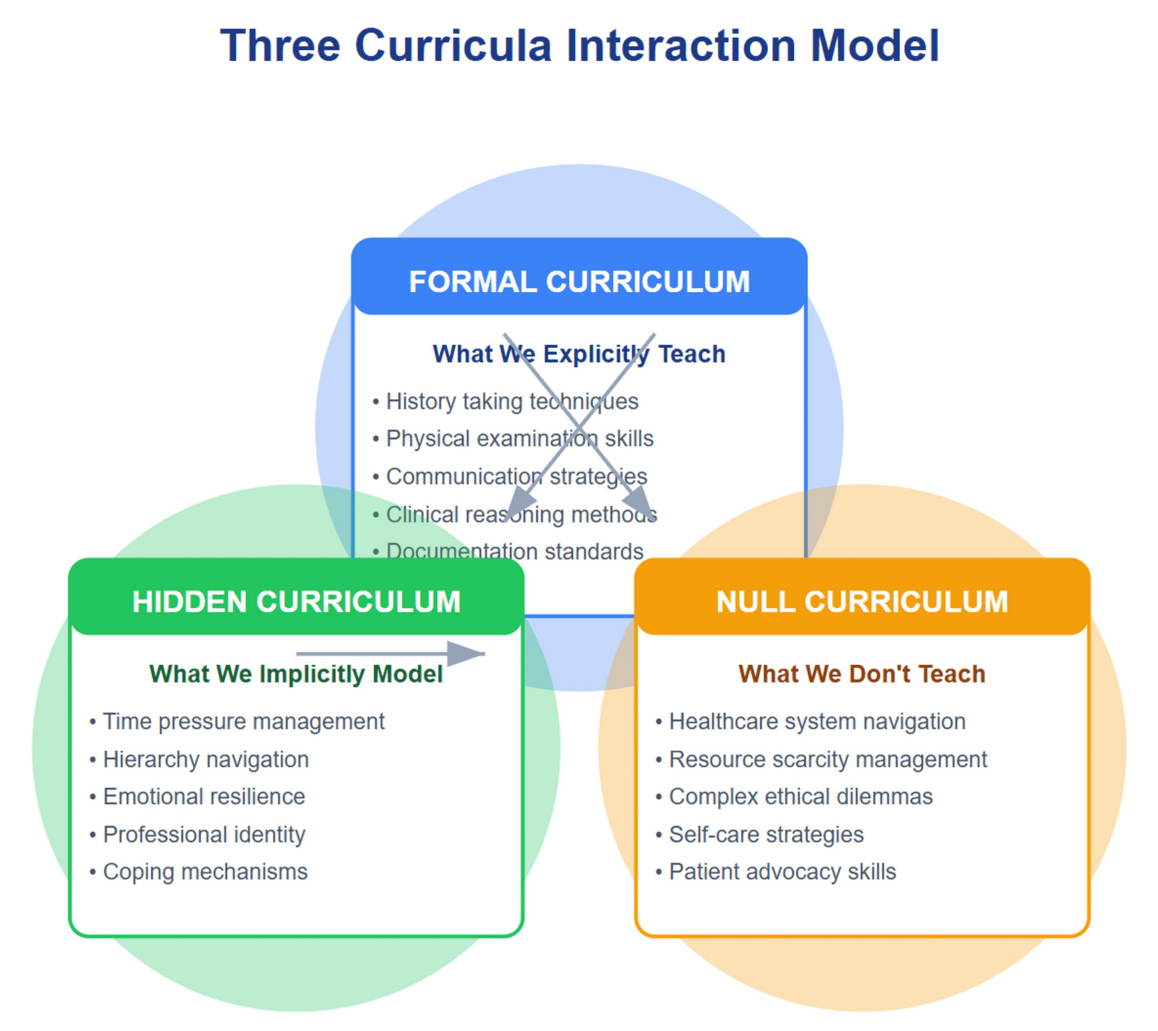
The three curricula framework Total student learning experience: integration of all three curricula shapes medical education outcomes. This image was created by the authors of this study.

This comprehensive clinical skills course considers the three types of curricula: formal, hidden, and null (Figure 4). The formal curriculum, outlined explicitly in the syllabus, covers essential content such as history taking, physical examination techniques, communication skills, and clinical reasoning. For example, students are taught structured approaches to interviewing, complete system-based physical examinations, and proper documentation using the patient-oriented medical record.

The hidden curriculum, learned through observation and experience, reinforces professionalism, teamwork, time management, and emotional intelligence [4]. These elements emerge naturally during small-group sessions, where students observe how instructors interact respectfully with standardized patients, manage time during encounters, and model behaviors such as empathy, maintaining patient dignity, and responding calmly in challenging interactions.

Meanwhile, the null curriculum, representing what is not explicitly taught, may include ethical dilemmas, navigating complex healthcare systems, and advocating for marginalized patients. For instance, while students learn how to take a history, they may not receive explicit instruction on addressing sensitive issues such as financial barriers to care, health inequities, or end-of-life communication, topics that remain outside the structured curriculum unless intentionally introduced.

These curricula interact to provide a holistic learning experience: the formal curriculum establishes foundational skills, the hidden curriculum demonstrates real-world application [5], and the null curriculum identifies areas requiring further attention. To enhance the course's effectiveness, we should make the hidden curriculum explicit, address the null curriculum through role-playing scenarios, and continuously update the formal curriculum to align with current medical practices. By incorporating all three curricula, this course equips medical students with the comprehensive skills necessary for successful patient care.

Balancing process and product: an integrated approach of clinical skills training

**Figure 5:**
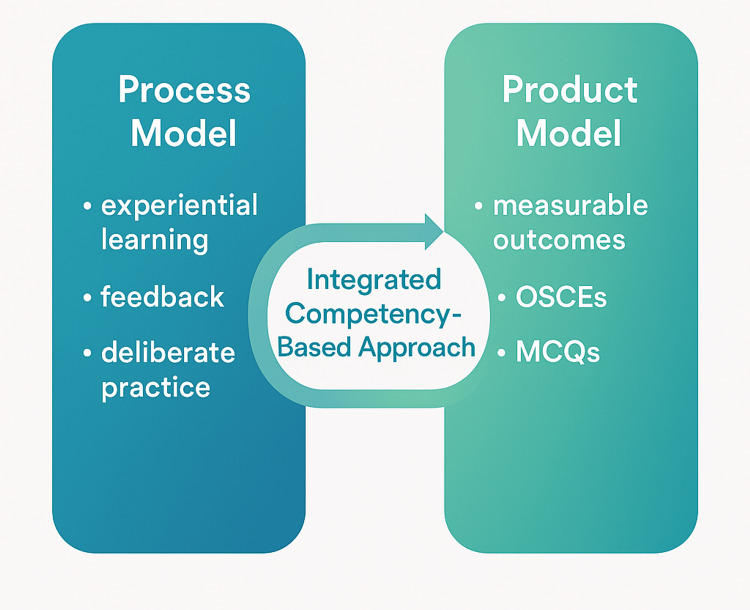
Balance between process and product models in clinical skills training OSCEs: Objective Structured Clinical Examinations; MCQs: Multiple-Choice Questions This image was created by the authors of this study.

This course incorporates elements of both a process model and a product model. The process model emphasizes skill development through experiential learning activities and engagement in various stages of skill acquisition. Within the clinical skills course, students are tasked with mastering competencies such as history taking, physical examination techniques, and effective communication through a structured series of instructional methods, including lectures, demonstrations, practice sessions, and feedback mechanisms. On the other hand, the product model emphasizes the attainment of specific outcomes or knowledge bases [6] (Figure 5). While there are definable knowledge components inherent in the clinical skills curriculum, such as understanding different examination techniques, the primary objective remains the proficient performance of these skills by students [7]. The integration of both models in the clinical skills course reflects a balanced approach that underscores the importance of both the process of skill acquisition and the ultimate goal of producing competent clinicians with a defined skillset.

Moreover, this course aligns with the principles of competency-based education (CBE). CBE focuses on the mastery of specific skills and knowledge rather than time spent in a course. In this curriculum, students are expected to demonstrate competency in various clinical skills and attitudes by the end of the course. The learning outcomes are clearly defined, and assessments are designed to measure students' proficiency in these competencies [8]. Additionally, the course emphasizes deliberate practice and repetitive learning activities to ensure skill acquisition, which is a hallmark of CBE. Therefore, while not explicitly labeled as such, the structure and approach of the clinical skills course reflect elements of CBE [7].

## Discussion

Strengths and potential areas for improvement

This course boasts several strengths that contribute to its effectiveness. Firstly, it features well-defined learning objectives and a structured curriculum, ensuring clarity and direction for learners. The flexible timetable accommodates diverse schedules, guaranteeing equal opportunities for all participants to engage in learning activities. Additionally, early exposure to interprofessional teams allows students to develop essential soft skills and gain insights into collaborative healthcare practices. Moreover, the course employs a variety of teaching methods, leveraging tools like the educational platform Moodle to provide a wealth of educational resources. These resources, including online resources, clinical protocols, forums for discussion, PowerPoint presentations, and instructional videos, enhance the learning experience and cater to diverse learning styles [9].

One strength is its integration with organ system-based courses, which can provide students with a contextual understanding of how clinical skills apply to different medical conditions. This approach allows for a more holistic learning experience and better prepares students for clinical practice. Another positive aspect is the emphasis on repetitive and deliberate practice to ensure skill acquisition [10]. This active learning approach is known to be effective in solidifying knowledge and skills, which can lead to better retention and application in real-world settings. Additionally, the inclusion of both formative and summative assessments, such as real-time feedback and OSCEs/MCQ exams, respectively, provides students with multiple opportunities to demonstrate their proficiency and receive constructive feedback for improvement. However, there are some potential limitations to consider. The course description mentions a focus on normal findings in the first part of the course, which may not adequately prepare students for encountering abnormal or complex cases in clinical practice. It would be beneficial to include training on recognizing and managing abnormal findings early in the curriculum [11].

Furthermore, while integration with organ system-based courses is beneficial, ensuring relevance and continuity in learning [12], there should be careful coordination to ensure that clinical skills instruction aligns effectively with the content being covered in those courses. This will prevent the duplication of efforts and ensure that students are receiving cohesive and comprehensive instruction.

There are however limitations to the organ-based curriculum in teaching these skills, particularly when students encounter patients with multi-organ illnesses or complex conditions [13]. To enhance medical interviewing and physical examination skills, content and reasoning should be integrated. Combining teaching of content knowledge with clinical reasoning skills helps students understand the "how" and "why" of medical procedures [14]. While the course emphasizes clinical reasoning skills, there could be further emphasis on applying these skills to a broader range of clinical scenarios beyond those specified in the learning outcomes. There should be a shift from an organ-based curriculum to one that includes common multi-organ medical problems, preparing students for real-world clinical encounters. This approach emphasizes the importance of understanding the content related to each organ system, including physiology, pathology, and pharmacology. The goal is to help students integrate discrete content and reasoning skills, providing a more robust foundation for their clinical education and future medical practice.

Weaknesses

One significant concern revolves around the summative assessment process, which can instill anxiety in students. For instance, OSCEs often serve as a performance snapshot, which may be favored by certain candidates but can exacerbate stress levels for others [15]. Moreover, the effectiveness of education heavily relies on faculty delivering the teaching, which may be influenced by their clinical commitments, leading to potential variations in instruction quality. In fact, the course educators come from various specialties, such as family medicine, pediatrics, anesthesiology, internal medicine, and obstetrics. Training them adequately before sessions presents a significant challenge. Adequate and fair compensation is another issue. In fact, students receiving weekly structured instruction by compensated general practitioners demonstrate improved performance in OSCEs [7]. Concerning faculty members at universities, one potential challenge is the considerable effort required from many individuals over several months to implement an OSCE. In medical schools, most lecturers juggle responsibilities in teaching, research, and clinical practice. Therefore, to adopt more advanced evaluation methods like the OSCE, there is a need for additional teaching staff and improved administrative support [16].

**Figure 6:**
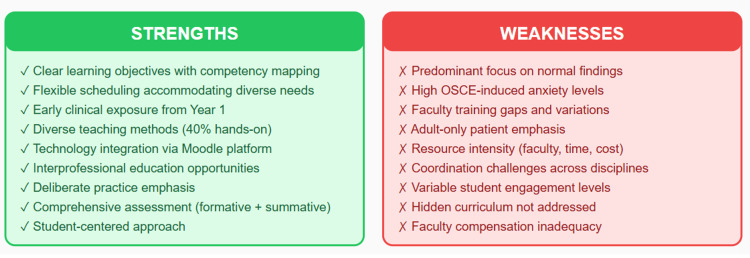
Strengths and weaknesses of the clinical skills course OSCE: Objective Structured Clinical Examination This image was created by the authors of this study.

**Figure 7:**
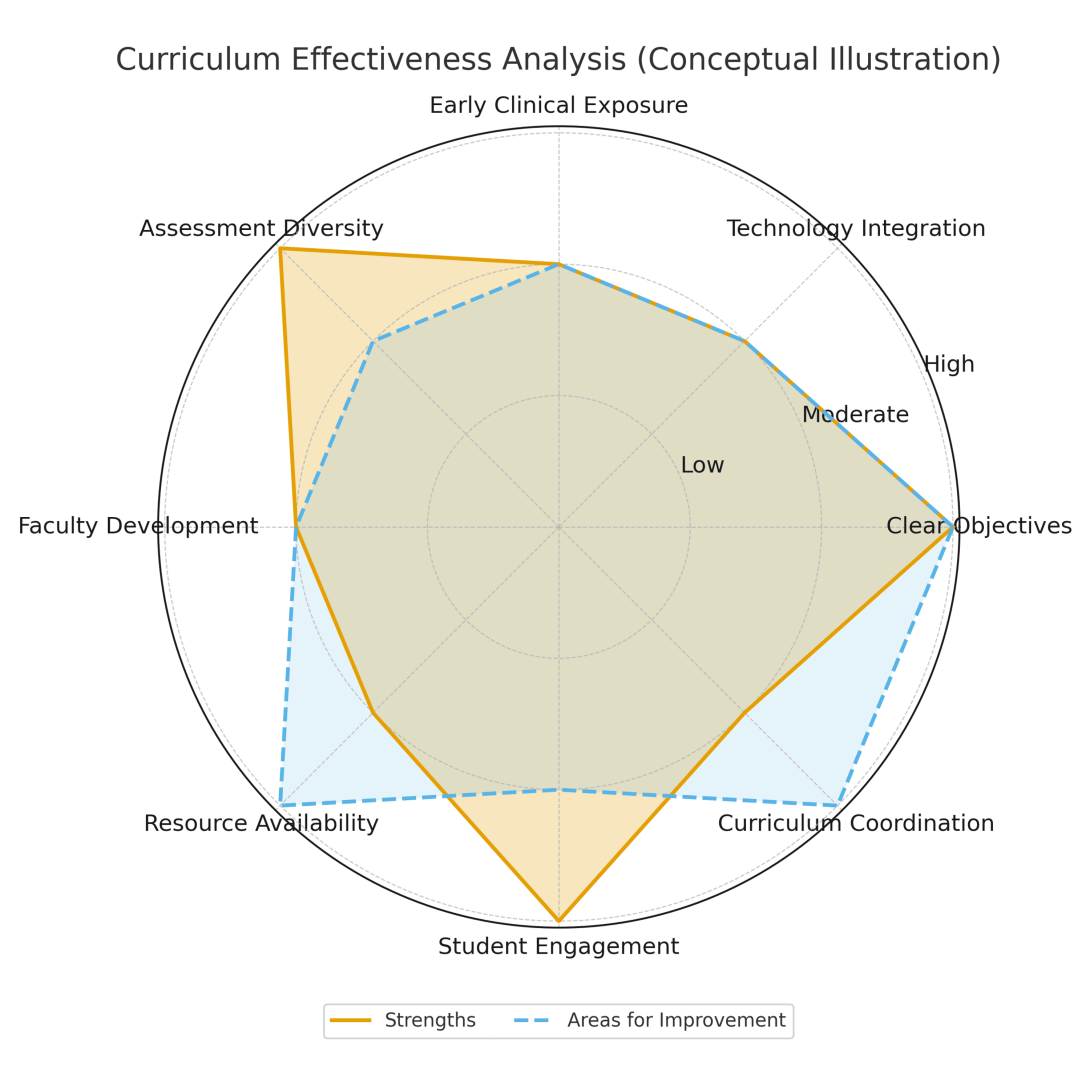
Conceptual curriculum strengths and areas for improvement A conceptual radar chart illustrating key strengths and areas for improvement in the clinical skills curriculum. Ratings (low/moderate/high) reflect qualitative judgment. This image was created by the authors of this study.

Additionally, the success of self-directed learning and group work hinges on student engagement, posing a challenge if participation levels are low. Attendance issues may further arise, as 10% of overall marks are often allocated for participation, potentially incentivizing attendance over genuine engagement. Furthermore, while students may be empowered with the prioritization of tasks, this may not necessarily align with the intended learning outcomes of the course. Finally, scheduling challenges may arise due to the flexibility of timetables, posing logistical hurdles for both students and faculty alike. Addressing these weaknesses necessitates a multifaceted approach that emphasizes student well-being, faculty support, and the alignment of assessment methods with learning objectives (Figure 6 and Figure 7). Potential strategies to mitigate these challenges include introducing low-stakes practice OSCEs and preparatory workshops to reduce assessment anxiety, developing standardized faculty training modules and providing protected teaching time to improve instructional consistency, and advocating for fair compensation or academic credit to enhance faculty engagement. Enhancing student participation may be achieved by incorporating clearer expectations within group work, shifting participation marks toward demonstrated preparation, and using learning analytics to identify disengaged learners. Additionally, aligning self-directed tasks with learning outcomes through structured milestone check-ins, and improving scheduling through centralized coordination systems or fixed teaching blocks, may help address logistical and organizational barriers.

Moreover, our course is mainly focused on adult patients. How about the pediatric population? How do we teach pre-clinical medical students how to examine and evaluate our smaller patients? It is technically unfeasible and unethical to bring real children as standardized patients. So here comes the role of simulation. Simulation-based education demands more resources and incurs higher costs compared to conventional methods.

Introducing a simulation-based curriculum during a pediatrics clerkship boosted knowledge scores and enhanced medical students' clinical performance throughout the clerkship. The American Association of Medical Colleges Task Force recommends a continuous clinical skills program for medical students. Although clinical skills are typically introduced in pre-clinical years, they are often neglected in later clinical training, particularly in specialized areas like pediatrics [17]. Teaching and ensuring clinical skill competence pose challenges in curriculum development. Traditionally, students learn through apprentice-style methods during clinical encounters, but they often lack observation and feedback. This deficiency leads to documented skill gaps among new doctors. Integrating newer educational methods, such as medical simulation, could improve pediatric clinical skills teaching. Simulation offers advantages like feedback, deliberate practice, outcome measurement, standardized exposure, and a safe learning environment. While simulation has proven effective in postgraduate education, its impact on undergraduate clinical clerkships, particularly in pediatrics, is still being explored.

Pros and cons of the OSCE

Evaluating clinical skills is central to medical education, and the OSCE is widely recognized as a valid, reliable, and efficient assessment method. In a typical OSCE, candidates rotate through stations staffed by authentic or standardized patients, performing clinical tasks evaluated with structured checklists. Although examinations are generally stressful, some evidence suggests that OSCEs may be less anxiety-provoking than traditional formats [15]. OSCEs provide a standardized means of assessing clinical reasoning, communication, and procedural skills, and OSCE performance correlates with other academic measures such as MCQ exams, supporting their relevance in student evaluation. To ensure fairness and reliability, educators should standardize scenarios, maintain quality control, provide detailed feedback, and regularly refine the examination process. Despite these advantages, OSCEs may correlate weakly with other assessments, may not fully capture cognitive knowledge, and often show wider score variability, which can influence student ranking [18].

The primary benefit of OSCEs, which is standardization, can be compromised without adequate planning or proper training for station designers, examiners, and standardized patients. Developing an OSCE requires extensive logistical preparation and coordinated training. Quality assurance measures, including post-examination psychometrics, help evaluate station performance and overall reliability, informing improvements for future administrations [19]. Implementing consistent faculty development programs may enhance acceptance of assessment innovations and promote a broader evaluation culture within the institution [15].

Curriculum evaluation

Because evaluation significantly contributes to enhancing the quality of educational programs, education policymakers should prioritize evaluating these programs and addressing any barriers and issues they face. To enhance program quality, policymakers and officials are encouraged to adopt the Context, Input, Process, and Product (CIPP) evaluation model. This systematic approach can assess all aspects of an educational program, from its development to its implementation.

**Figure 8:**
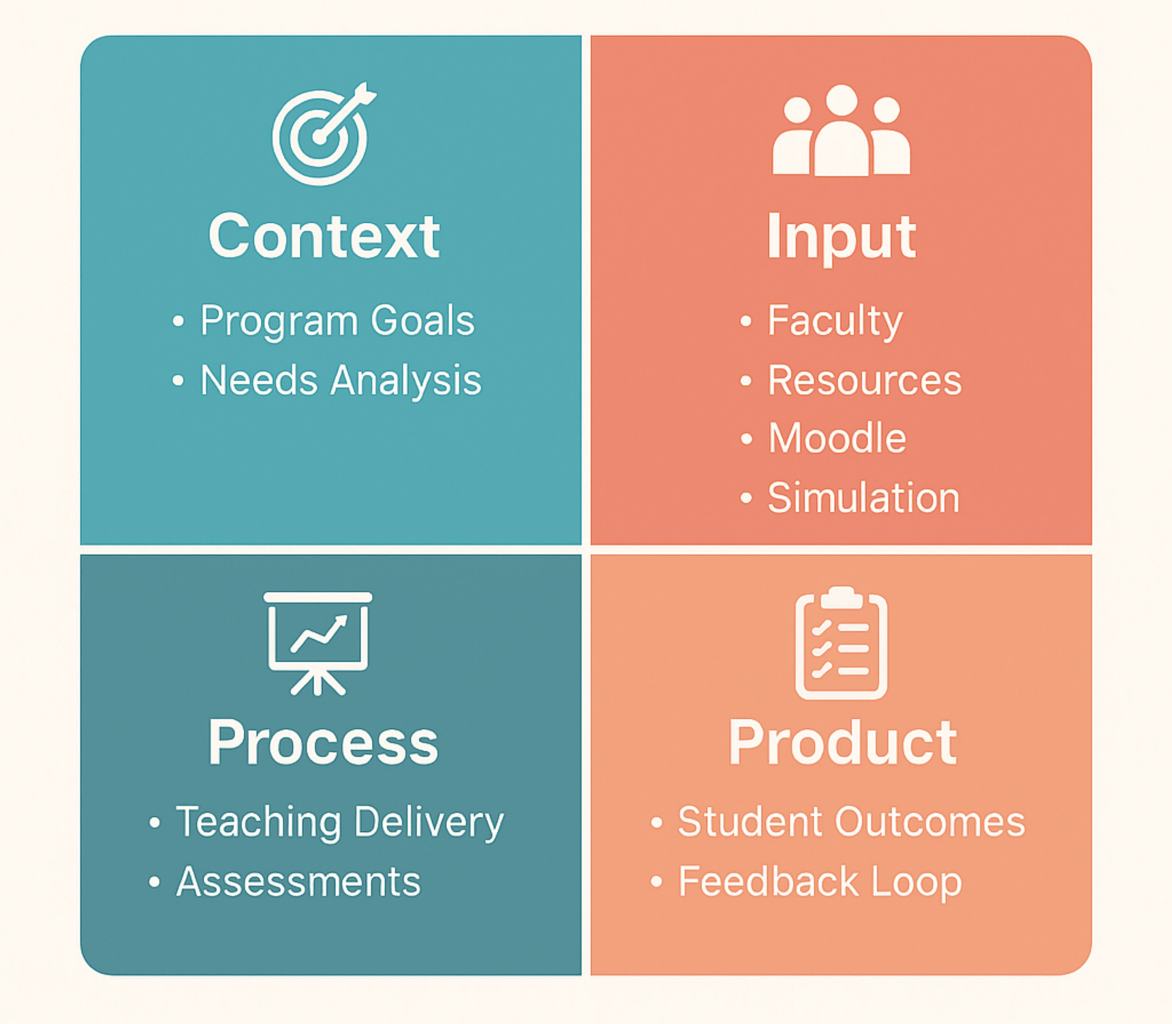
CIPP evaluation The CIPP evaluation framework applied to the clinical skills course. CIPP: Context, Input, Process, and Product This image was created by the authors of this study.

Improving the quality of higher education is crucial for a country's sustainable development. Achieving quality education involves adhering to appropriate standards and employing research and evaluation. Evaluation is essential for judging and documenting educational program quality and assessing its development and implementation. It helps understand program compatibility with individual and community needs and identifies factors for improvement. Principled evaluation forms the basis for educational decisions and plans, enhancing academic standards. The CIPP evaluation model, which assesses Context, Input, Process, and Product, provides a comprehensive framework for evaluating educational programs (Figure 8). Context evaluation establishes educational goals and identifies challenges and opportunities. Input evaluation focuses on resources and strategies for program implementation. Process evaluation monitors program implementation and its impact on learners. Output evaluation assesses program effectiveness and alignment with goals. Applied to the clinical skills course, Context evaluation examines the competencies expected of pre-clinical students and the gaps identified by faculty in previous clerkships. Input evaluation considers available resources, including trained instructors, simulation tools, standardized patients, and the integration of clinical skills sessions within the organ-system curriculum. Process evaluation assesses how well teaching sessions, OSCE preparation activities, feedback mechanisms, and small-group interactions are delivered, as well as student engagement throughout the course. Product evaluation analyzes outcomes such as OSCE performance, achievement of learning objectives, student confidence entering clerkships, and feedback indicating areas requiring refinement. Overall, evaluation based on the CIPP model aims to enhance program performance [20].

## Conclusions

The clinical skills course presents a comprehensive curriculum aimed at equipping students with foundational clinical skills and attitudes essential for medical practice. By progressively increasing the complexity of sessions and emphasizing repetitive practice, the curriculum facilitates skill acquisition effectively. The inclusion of both formative and summative assessments, including real-time feedback and OSCEs, allows for ongoing evaluation and ensures students' readiness for clinical practice. Overall, while this clinical skills curriculum has several positive elements, there are opportunities for enhancement, particularly in ensuring early exposure to abnormal findings and optimizing coordination with organ system-based courses. Regular evaluation and refinement of the curriculum based on student feedback and outcomes data will be essential for continuous improvement.
